# A Single-Substrate Biosensor with Spin-Coated Liquid Crystal Film for Simple, Sensitive and Label-Free Protein Detection

**DOI:** 10.3390/bios11100374

**Published:** 2021-10-06

**Authors:** Po-Chang Wu, Chao-Ping Pai, Mon-Juan Lee, Wei Lee

**Affiliations:** 1 Institute of Imaging and Biomedical Photonics, College of Photonics, National Yang Ming Chiao Tung University, Guiren Dist., Tainan 711010, Taiwan; jackywu@nycu.edu.tw (P.-C.W.); pdxdydz@gmail.com (C.-P.P.); 2 Department of Bioscience Technology, Chang Jung Christian University, Guiren Dist., Tainan 711301, Taiwan; 3 Department of Medical Science Industries, Chang Jung Christian University, Guiren Dist., Tainan 711301, Taiwan

**Keywords:** liquid crystal, spin-coating, single-substrate, label-free biosensor, bovine serum albumin, cancer biomarker CA125

## Abstract

A liquid crystal (LC)-based single-substrate biosensor was developed by spin-coating an LC thin film on a dimethyloctadecyl[3-(trimethoxysilyl)propyl]ammonium chloride (DMOAP)-decorated glass slide. Compared with the conventional sandwiched cell configuration, the simplified procedure for the preparation of an LC film allows the film thickness to be precisely controlled by adjusting the spin rate, thus eliminating personal errors involved in LC cell assembly. The limit of detection (LOD) for bovine serum albumin (BSA) was lowered from 10^−5^ g/mL with a 4.2-μm-thick sandwiched cell of the commercial LC E7 to 10^−^^7^ g/mL with a 4.2-μm-thick spin-coated E7 film and further to 10^−^^8^ g/mL by reducing the E7 film thickness to 3.4 μm. Moreover, by exploiting the LC film of the highly birefringent nematic LC HDN in the immunodetection of the cancer biomarker CA125, an LOD comparable to that determined with a sandwiched HDN cell was achieved at 10^−8^ g/mL CA125 using a capture antibody concentration an order of magnitude lower than that in the LC cell. Our results suggest that employing spin-coated LC film instead of conventional sandwiched LC cell provides a more reliable, reproducible, and cost-effective single-substrate platform, allowing simple fabrication of an LC-based biosensor for sensitive and label-free protein detection and immunoassay.

## 1. Introduction

Liquid crystals (LCs) have been extensively exploited as a sensing element for biological detections since Abbott et al. first demonstrated in 1998 the use of the well-known single compound LC 5CB to transduce and amplify the optical signal produced by ligand‒receptor binding at LC‒solid interfaces for the detection of avidin [1]. By virtue of the unique properties of LCs, including optical anisotropy, fast stimuli-responsive molecular orientation, long-range anchoring transition and short-range intermolecular interaction, the LC-based biosensing mechanism is based on the presence of proteins or biological binding events at the interface (e.g., LC‒aqueous or LC‒solid) that is capable of reorienting LC molecules, typically from a uniform homeotropic or planar state to a disrupted state [2]. Such a response in LC orientation can then be transduced into a visible optical signal to the naked eye by observing the LC texture under crossed polarizers, allowing label-free detection to be achieved with the advantages of high sensitivity, rapid response, low cost, and simple operation. A wide variety of LC-based biosensing technologies were demonstrated at the LC‒solid or LC‒aqueous interface for different types of biological analytes, with strategies to improve sensing performance summarized in several review papers [3,4,5,6,7,8,9]. While conventional optical texture observation mainly permits qualitative analysis of the optical signal, attention has been paid to developing quantitative biosensing techniques by exploiting the dielectric and electro-optical characteristics of nematic LCs [10,11,12], Bragg reflection of chiral LCs [13,14,15], and selective absorption features of a dye-LC as well as a dye-doped LC [16,17].

Because of its fluidity, LC is typically confined in a well-defined compartment for biosensing applications. Conventionally, an LC‒aqueous interface is established by filling LCs in a transmission electron microscopy (TEM) grid mesh, with homeotropic and planar anchoring at the LC‒air and LC‒water interface, respectively. In the solid‒LC‒aqueous configuration, the TEM grid was placed on a glass substrate coated with silane coupling agents (e.g., dimethyloctadecyl[3-(trimethoxysilyl)propyl]ammonium chloride, DMOAP or octadecyltrichlorosilane, OTS) and impregnated with LC, followed by immersion in an aqueous solution containing the analyte [18,19], while the air‒LC‒aqueous configuration was constituted by layering the LC-filled TEM grid on top of and in contact with the aqueous phase, keeping the other side of the LC film exposed to the air [20,21]. On the other hand, most biosensing platforms relying on detection at the LC‒solid interface are developed with a sandwiched LC cell configuration, in which the LC is enclosed between a pair of glass substrates with the inner surfaces coated with DMOAP or OTS to support homeotropic anchoring of LC molecules. Therefore, the presence of biomolecules on one of the substrate surfaces can be detected with high signal-to-background contrast by the dark-to-bright transition of the optical LC texture when the homeotropic alignment is disrupted [22]. However, fabrication of an LC-based biosensor with a sandwiched LC cell or LC-filled TEM grid is time-consuming and requires trained personnel, and procedures such as the construction of an LC cell (e.g., spacer dispersion, adhesive sealing, and substrates assembly) may introduce personal errors in the uniformity and thickness of the LC layer, which would reduce sample-to-sample reproducibility as well as accuracy and reliability of an LC-based bioassay. As such, LC-based sensing platforms eliminating cell assembly have been proposed, such as injecting LCs in microfluidic channels [23,24] or rectangular capillaries [25] for the detection of antibody‒antigen immunobinding on a solid surface or dispensing LC-in-water droplet patterns on an OTS-treated substrate for detection in an aqueous solution [26,27].

Along the line of simplifying the procedure of fabrication, in this study we proposed to spin-coat LCs in the form of a thin film directly on a DMOAP glass substrate and exploited this single-substrate configuration as the sensing platform to report the presence of protein or the occurrence of specific antibody‒antigen interactions on the solid surface. Spin-coating is a mature manufacturing process in the microelectronics and semiconductor industries that utilizes the centrifugal force to simply and rapidly deposit a thin film on a flat surface with thickness ranging from micro- to nanometers, which is controllable by adjusting the spin rate. So far, the spin-coating technique has been widely applied to support film formation of photo-resistant, polymeric, and semiconducting materials for optical, electronic, solar cell, semiconducting, display, and sensing applications. In contrast to traditional sandwiched LC cells, the spin-coating procedure can be easily familiarized by an inexperienced user to obtain an LC film. Spin-coated LC film facilitates fundamental investigations on LC phase transition and morphological transformation [28,29], as well as light-driven helical rotation and pitch tuning of cholesteric LC gratings [30]. In our previous studies, the single-substrate biosensing technology was reported with spin-coated films of an LC-photopolymer composite and cholesteric LC [15,31]. Herein, we extend the application of single-substrate detection to nematic LC films in protein assay with bovine serum albumin (BSA) as the protein standard and immunoassay of the cancer biomarker CA125. Results were compared with those using a sandwiched LC cell and approaches to signal amplification concerning LC film thickness were discussed.

## 2. Materials and Methods

### 2.1. Materials

The NEG AT35 optical glass slides with the dimensions of 22 mm in length, 18 mm in width and 1.1 mm in thickness were received from Ruilong Glass, Taiwan. The aligning agent DMOAP was purchased from Sigma-Aldrich (St. Louis, MO, USA). The protein standard BSA (Sigma-Aldrich, St. Louis, MO, USA) with a molecular weight of 66.43 kDa was adopted in protein assays, while recombinant human CA125 (MUC16) protein received from R&D Systems (Minneapolis, MN, USA) and anti-CA125 antibody provided by Santa Cruz Biotechnology (Dallas, TX, USA) were used in immunoassays. The two eutectic nematic LCs, E7 and HDN, were obtained from Daily Polymer Co., Taiwan, and Jiangsu Hecheng Display Technology Co., China, respectively. Their clearing temperatures (*T*_c_) are 58 °C and 97 °C, while birefringence (Δ*n*) measured at the wavelength of 589 nm and temperature of 20 °C is 0.225 and 0.333 for E7 and HDN, respectively. Deionized (DI) water, purified by an RDI reverse osmosis/deionizer system, was used to prepare all aqueous solutions.

### 2.2. Formation of DMOAP Monolayer

DMOAP-coated substrates were prepared to bear immobilized biomolecules (e.g., BSA and anti-CA125 antibodies) and to support homeotropic LC orientation at the LC‒glass interface. Following the procedure for cleaning purposes established in our previous works, optical glass slides prior to use in experiments were washed under ultrasonication with a sequence of an aqueous solution of detergent, DI water and 99% ethanol. After performing each of the above-mentioned steps at room temperature for 15 min, cleaned glass slides were dried with nitrogen, baked in an oven at 74 °C for 15 min and then cooled down naturally to room temperature. Using the dip-coating method, cleaned glass slides were immersed and ultrasonicated in an aqueous solution containing 1% (*v*/*v*) DMOAP for 15 min. After washing in DI water for 15 min and drying under a stream of nitrogen, these slides were baked in an oven at 85 °C for 15 min to form a stable and uniform DMOAP monolayer on the glass surface.

### 2.3. Immobilization of BSA

Aqueous solutions with designated BSA concentrations were prepared by serial dilution of a BSA stock solution with DI water. A DMOAP-coated glass slide was dispensed with four droplets (5 μL/droplet) of BSA solution at a given concentration using a micropipette to form a 2 × 2 protein array. After incubation at 30 °C for 1 h to allow immobilization of BSA, the slide was rinsed twice with DI water to remove unbound BSA and then incubated in an oven at 30 °C for 30 min to allow evaporation of DI water without affecting BSA activity.

### 2.4. Specific Binding of Anti-CA125 Antibody to the CA125 Antigen

Anti-CA125 antibody was diluted to the desired concentration with DI water, while the lyophilized powder of CA125 was reconstituted in phosphate buffered saline followed by serial dilution in DI water. To perform a CA125 immunoassay, anti-CA125 antibody was immobilized at 5 μL/droplet on a DMOAP-coated substrate in a 2 × 2 array format. After drying for 1 h and rinsing twice thoroughly with DI water, the substrate was dispensed with 15 μL CA125 antigen and covered with a cleaned cover glass to allow specific binding of CA125 to the immobilized anti-CA125 antibody for 30 min. After removing the cover glass, the substrate was rinsed twice with DI water to eliminate unbound CA125 and then dried in an oven at 30 °C.

### 2.5. LC Cell Assembly and Spin-Coating of LC Films

An LC cell was fabricated by assembling a BSA-immobilized substrate and another BSA-free DMOAP-coated slide, with the DMOAP coating and immobilized BSA facing inward, to form a sandwiched cell of 4.2 ± 0.5 μm (determined by the size of ball spacers) in cell gap, which was then filled with LC through capillary force. LC films were formed by dispensing 5 μL of E7 or HDN on areas on the glass substrate immobilized with the analyte, followed by spin-coating with a SP-D_3_-P spin coater (APISC Corp., Taiwan), which determines the number of steps and the corresponding spin rates and duration. For the protein assay, LC films of E7 were formed on BSA-immobilized substrates by a default three-step spin-coating program of 500 rpm for 10 s, and consecutively 1000 rpm for 10 s and 3000 rpm for 10 s, while for the CA125 immunoassay, LC films of E7 or HDN were formed in a single-step spin-coating procedure at 5000 rpm for 20 s. All spin-coated LC films can be uniformly and stably preserved without shrinkage for several hours at room temperature to allow texture observation or optical measurements to be accomplished.

### 2.6. Optical LC Texture Observation and Measurement of LC Film Thickness

**Figure 1:**
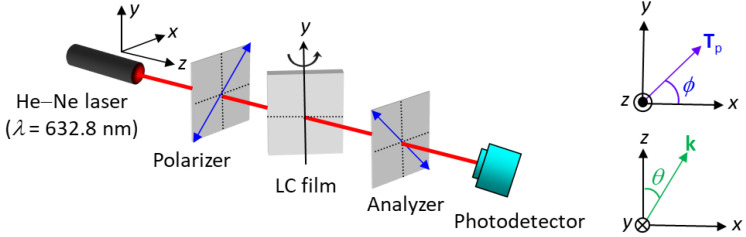
Schematic of the optical setup with crossed polarizers for the measurement of the thickness of LC films spin-coated on a DMOAP-coated glass substrate.

All measurements were carried out at room temperature at which LCs were in the nematic phase. Optical textures of spin-coated LC films and sandwiched LC cells were observed under crossed polarizers using an Olympus BX51 polarizing optical microscope (POM) in the transmission mode with a 4× objective lens. Microscopic images were taken by an Olympus XC30 digital camera mounted on the microscope with a resolution of 2080 × 1544 pixels and an exposure time of 100 ms. The thickness of the spin-coated LC film was determined by the optical setup as shown in Figure 1. A sample consisting of a DMOAP-coated substrate spin-coated with an LC film was placed between a polarizer and an analyzer whose transmission axes were perpendicular to each other. The incident light source was a He-Ne laser with an emission wavelength of *λ* = 632.8 nm. In this manner, because the LC film spin-coated on a DMOAP substrate exhibits homeotropic alignment, it can be regarded as a uniaxial crystal film with the optic axis perpendicular to the film plane (i.e., *x*-*y* plane), and the correlation between the transmittance (*I*) of light received by the detector and the angle of light incidence (*θ*) can be specified as

(1) $$I=\sin^{2}(2\phi )\sin^{2}\left[\frac{\pi n_{o}d_{\mathrm{LC}}}{\lambda }\left(\sqrt{1-\frac{\sin^{2}\theta }{n_{e}^{2}}}-\sqrt{1-\frac{\sin^{2}\theta }{n_{o}^{2}}}\right)\right]$$

where *d*_LC_ is the LC film thickness, *n*_e_ and *n*_o_ are parallel and perpendicular components of the refractive index of LC, respectively, *θ* is the (polar) angle between the direction of propagation of light (i.e., the *z-*axis) and the substrate normal (**k**), namely, the unperturbed LC director lying in the *x*-*z* plane, and *Φ* is the angle between the transmission axis of the polarizer (**T**_p_) and the *x*-axis [32]. It should be emphasized that the second term in the brackets of Equation (1), estimated based on the law of refraction and the index ellipsoid equation [32], is valid and specific to the phase retardation of a vertically aligned LC film at an arbitrary light incident angle. The value of *d*_LC_ was deduced from Equation (1) by fitting the measured dependence of *I* on *θ*. Note that the conventional interference method to obtain LC film thickness was not applicable in this study because the perpendicular component of the refractive index of LCs (e.g., *n*_o_ = 1.52 for E7 and *n*_o_ = 1.51 for HDN) is close to that of the glass substrate.

## 3. Results and Discussion

### 3.1. LC-Based Single-Substrate Protein Assay with Spin-Coated E7 Film

**Figure 2:**
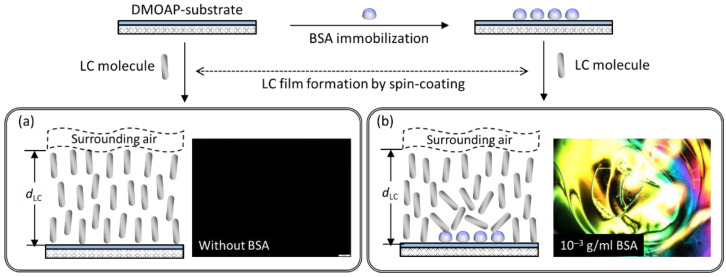
Schematic of the working principle of the LC-based single-substrate protein assay for the detection of BSA with spin-coated LC film on a DMOAP-coated substrate. (**a**) In the absence of BSA, LC molecules are aligned homeotropically, resulting in uniformly dark optical texture. (**b**) In the presence of BSA, LC alignment is disrupted at the LC‒glass interface, giving rise to non-uniform and bright optical texture.

The LC-based single-substrate protein assay was developed by immobilizing the protein standard BSA on a DMOAP-coated substrate, followed by detection with spin-coated LC film, as depicted in Figure 2. Due to the homeotropic anchoring strength provided by both DMOAP and the air, LC molecules in the semi-free LC film were vertically anchored with their long axes perpendicular to the substrate plane at the LC‒DMOAP and LC‒air interfaces. A uniformly dark optical LC texture was obtained in the absence of BSA because no phase retardation was experienced when the normally incident polarized light passed through the homeotropically aligned LC film (Figure 2a). When the LC film was spin-coated on a BSA-immobilized DMOAP substrate, LC molecules were reoriented from the homeotropic to the disrupted state. Consequently, the birefringence effect and light scattering caused the appearance of bright but non-uniform optical texture under crossed polarizers (Figure 2b).

**Figure 3:**
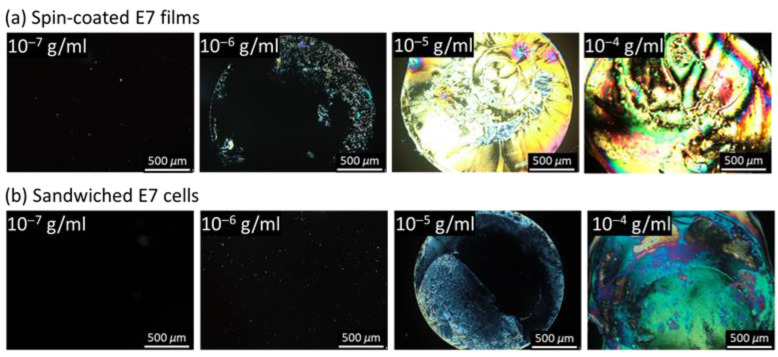
Polarized optical textures of E7 in spin-coated films and LC cells in the presence of BSA. The nematic LC E7 was (**a**) spin-coated as a thin film on a DMOAP-coated glass substrate or (**b**) sandwiched between two glass substrates in an LC cell at various BSA concentrations ranging from 10^−7^ to 10^−4^ g/mL. Scale bar, 500 μm.

Figure 3 compares the optical textures of spin-coated E7 films and sandwiched E7 cells at BSA concentrations (*c*_BSA_) between 10^−7^ and 10^−4^ g/mL. Here, the thickness of the E7 film formed by the spin-coater under default conditions described in Section 2.5 was *d*_LC_ ~5.5 μm, while that in the LC cell was 4.2 ± 0.5 μm. As shown in Figure 3a for spin-coated E7 films on BSA-immobilized DMOAP substrates, the optical texture was uniform with a dark appearance at *c*_BSA_ = 10^−7^ g/mL but became non-uniform with bright domains in the dark background at *c*_BSA_ = 10^−6^ g/mL. Increasing the BSA concentration further to *c*_BSA_ = 10^−5^ and 10^−4^ g/mL resulted in brighter textures than that at *c*_BSA_ = 10^−6^ g/mL. The limit of detection (LOD) is thus of the order of magnitude of 10^−6^ g/mL, meaning that the amount of immobilized BSA at concentrations lower than 10^−6^ g/mL may be insufficient to weaken the anchoring strength of DMOAP and, in turn, to disrupt the homeotropic orientation of E7. On the other hand, the dark-to-bright optical response to BSA occurred at 10^−5^ g/mL when detected with the sandwiched E7 cell (Figure 3b). At *c*_BSA_ = 10^−6^ g/mL or lower, a dark texture similar to that in the absence of BSA was observed. These results indicate that using spin-coated LC film instead of the conventional sandwiched LC cell as the sensing platform for protein assay not only simplified the fabrication procedure but enhanced detection sensitivity. This can be explained by the weaker anchoring strength at the LC‒air interface compared with that at the LC‒DMOAP interface so that LC molecules in the LC film are more easily disrupted in the presence of BSA than those in the LC cell.

### 3.2. Signal Amplification of Single-Substrate Detection through the Control of Film Thickness

**Figure 4:**
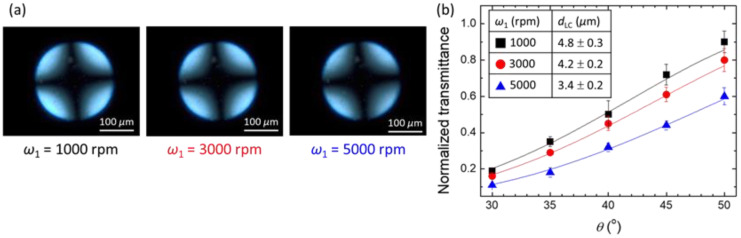
Correlation between spin rate and LC film thickness. The E7 film was spin-coated on DMOAP substrates with a two-step procedure in which the spin rate of the first step *ω*_1_ = 1000, 3000 or 5000 rpm and that of the second step *ω*_2_ = 5000 rpm, with each step lasting for 10 s. (**a**) Conoscopic images of LC films spread at various *ω*_1_ observed under a POM. Each error bar denotes the standard deviation calculated from the transmission values of five independent measurements. (**b**) Dependence of transmittance on incident angle of light *θ* measured with the optical setup in Figure 1. The values of E7 film thickness displayed in the inset of (**b**) were deduced by fitting the experimental results with Equation (1).

The thickness of an LC film can be directly and accurately controlled by adjusting the spin rate. The correlation between the spin rate and LC film thickness was demonstrated with a two-step spin-coating procedure in which the spin rate of the first step (*ω*_1_) was varied from 1000 to 5000 rpm, while that in the second step (*ω*_2_) was fixed at 5000 rpm, with the duration of both steps set at 10 s (Figure 4). The conoscopic image and the average thickness of an E7 film and the corresponding uncertainty were evaluated from five independent sets of experiments to ensure the reproducibility. The homeotropic alignment of the spin-coated E7 film with *ω*_1_ = 1000 rpm, 3000 rpm, or 5000 rpm on a DMOAP substrate was confirmed by the Maltese cross pattern observed in conoscopic images (Figure 4a). The thickness of a spin-coated LC film was determined by measuring optical transmittance at various incident angles of light (*θ*) with respect to the substrate normal based on the optical setup in Figure 1. As shown in Figure 4b, the transmittance of the LC film increased with increasing *θ* from 30° to 50°. Because the phase retardation of light passing through an LC film at *θ* < 50° is lower than *π*/2, thicker LC films resulted in higher transmittance at a fixed *θ*, according to Equation (1). As a result, by fitting the experimental data in Figure 4b with the equation, the LC film thickness can be deduced as *d*_LC_ = 4.8 ± 0.3 μm at *ω*_1_ = 1000 rpm, *d*_LC_ = 4.2 ± 0.2 μm at *ω*_1_ = 3000 rpm, and *d*_LC_ = 3.4 ± 0.2 μm at *ω*_1_ = 5000 rpm. Note that the uncertainty of the film thickness of ~±0.2 μm is obtained by calculating the standard deviation from five thickness values. This result supports that the LC film can be readily formed on a solid surface with high controllability and reproducibility by using the conventional spin-coating method.

**Figure 5:**
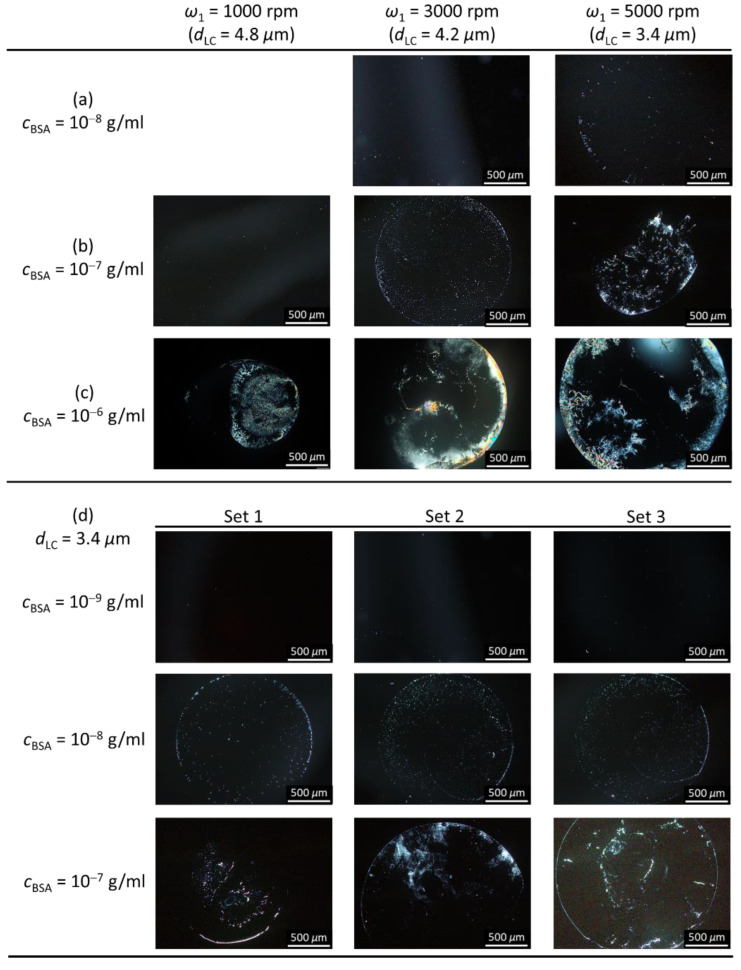
Polarized optical textures of spin-coated E7 films of various thicknesses in the presence of BSA. DMOAP-coated glass substrates with immobilized BSA at concentrations of (**a**) 10^−8^, (**b**) 10^−7^, and (**c**) 10^−6^ g/mL were spin-coated with the nematic LC E7 by varying the spin rate of the first step (*ω*_1_) of a two-step spin-coating procedure to form LC films with thicknesses *d*_LC_ of 4.8, 4.2, and 3.4 μm at *ω*_1_ = 1000, 3000, and 5000 rpm, respectively. (**d**) Optical textures of three independent sets of experiments for 3.4 μm-thick E7 films in the presence of BSA. Note that the brightness of optical textures in Figure 5a,b,d has been artificially increased by 40% to enhance the visibility. Scale bar, 500 μm.

We further investigated the effect of LC film thickness on the LOD for BSA in the proposed LC-based single-substrate protein assay. Here, the brightness of optical images at *c*_BSA_ = 10^−8^ g/mL (Figure 5a) and 10^−7^ g/mL (Figure 5b) has been artificially increased by 40% to enhance the visibility of bright domains. At a BSA concentration of 10^−7^ or 10^−6^ g/mL, it is demonstrated in Figure 5b and c that the bright domains in the optical texture increased as the film thickness decreased from 4.8 to 3.4 μm. Notably, when BSA concentration was lowered to 10^−8^ g/mL a trace amount of light leakage was still discernible in the optical texture of the 3.4-μm-thick E7 film, while the optical texture was completely dark for the 4.2-μm-thick film (Figure 5a). Good reproducibility of the LOD for BSA detection with spin-coated E7 films was ascertained from the reproducible results at least in four of the five same experiments. For example, in the case of 3.4-μm-thick E7 films spin-coated on BSA-immobilized substrates, consistent results can be obtained from another three sets of experiments at BSA concentrations of 10^−7^–10^−9^ g/mL, including determinable LOD on the order of 10^−8^ g/mL from dark-to-bright change in the optical image and increasing brightness and bright domains with increasing BSA concentration (Figure 5d). Note that the appearances of repeated optical images were different in general because BSA molecules were free to become immobilized at any place of a given area. The improved LOD for the thinner LC film suggests that the extent of disruption in LC alignment by BSA at the LC‒DMOAP interface can be enhanced by reducing the thickness of spin-coated LC films, thus leading to signal amplification. The thickness of LC cells has long been associated with the electro-optical performance of LC display devices. A thin cell exhibits stronger surface interaction and is expected to offer higher image intensity compared with a thick counterpart [7]. Nevertheless, the relevance of LC film thickness to signal amplification in LC-based biosensing has not been implicated until this study, presumably due to the lack of flexibility in controlling the thickness of the LC film. For instance, the LC film formed on an LC–aqueous interface is usually fixed to ~20 μm, conditioned by the thickness of the TEM grid. For manually assembled LC cells, the LC film thickness is determined by the spacer and is typically around 5–10 μm. A smaller cell gap for the LC cell can be achieved but may compromise the uniformity of the sandwiched LC layer and the reproducibility of signal output. Taking advantage of spin-coating, LC film thickness can be directly and easily reduced to a desired smaller value (<5 μm) to enhance the optical response.

Signal amplification mediated by labeling with gold nanoparticles has been reported in several LC-based biosensors [33,34,35,36,37]. To eliminate the costly and time-consuming procedure of labeling, a number of label-free approaches aimed at enhancing the optical signal and thereby improving detection sensitivity at the LC‒solid interface of a sandwiched LC cell were proposed, which include the use of a highly birefringent LC [38,39] and LC‒photopolymer composite [12], adjustment of the direction of linearly polarized light for a dye-doped LC [16], modification of the alignment layer by ultraviolet light irradiation [40], and application of a weak electric field to orient LC molecules in a pre-tilted state [41]. Because of the similar working principles between the LC film and LC cell in biodetection at the LC‒glass interface, most of the previously reported signal amplification approaches for sandwiched cells would be applicable to the LC film. We have demonstrated that signal amplification can be achieved with both spin-coated film and sandwiched cell of an LC‒photopolymer composite [12,31]. In addition, the intensity of the optical response to BSA was enhanced when a 3.4 μm-thick E7 film was spin-coated on DMOAP slides modified with ultraviolet light or when the high-birefringence LC HDN was used instead of E7 to form the sensing LC film, resulting in an improvement in LOD from 10^−8^ to 10^−9^ g/mL BSA (data not shown). In addition to LC–solid interface sensing, a few works have been conducted at the LC–aqueous interface for BSA detection with LOD of ~45 nM using the typical LC-infiltrated TEM grids configuration [42,43]. Notably, it is of perspective to extend the proposed LC-on-a-single-substrate configuration to implementation of protein assay at the LC–aqueous interface because the side open to the air can form an LC–aqueous interface analogous to that of LC-infiltrated TEM grids.

### 3.3. LC-Based Single-Substrate Immunoassay for CA125 with Spin-Coated E7 or HDN Film

**Figure 6:**
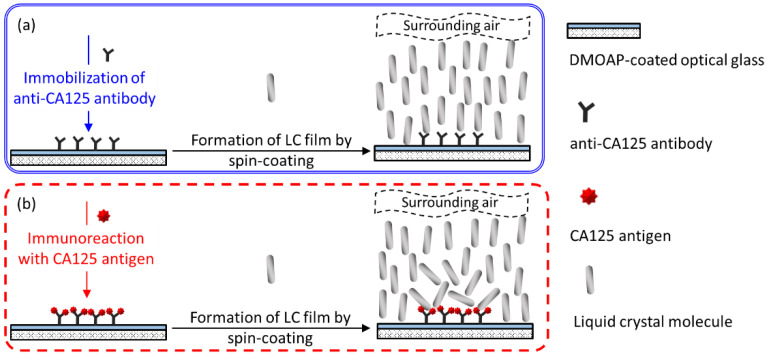
Schematics of the working principle of the LC-based single-substrate immunoassay for the detection of CA125 with spin-coated LC film on a DMOAP-coated substrate. (**a**) In the absence of CA125, LC molecules are aligned homeotropically on immobilized anti-CA125 antibody, whose amount is adjusted to a maximum without disturbing the orientation of LCs. (**b**) In the presence of CA125, alignment of LC molecules is disrupted due to the formation of the CA125 immunocomplex atop the DMOAP aligning layer.

**Figure 7:**
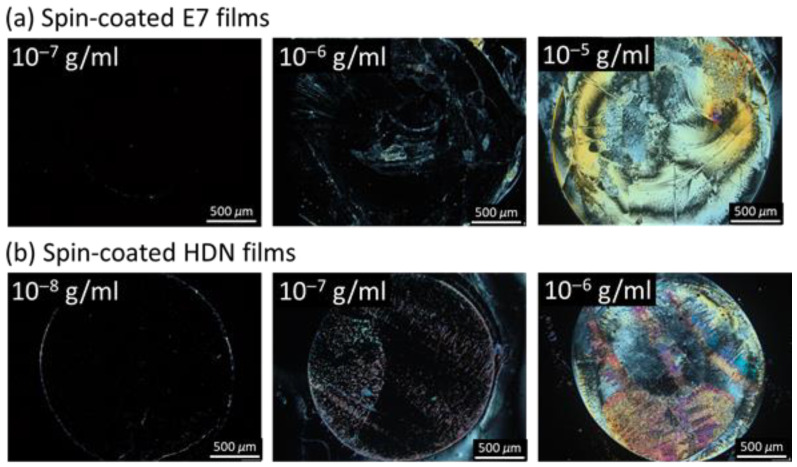
Polarized optical textures of spin-coated E7 and HDN films in the presence of the anti-CA125 antibody. The nematic LCs (**a**) E7 and (**b**) HDN were spin-coated on DMOAP-coated substrates immobilized with the anti-CA125 antibody at concentrations ranging from 10^−8^ to 10^−5^ g/mL. Scale bar, 500 μm.

As illustrated in Figure 6, the sensing principle of an LC-based single-substrate immunoassay depends on the specific binding of the CA125 antigen to anti-CA125 antibody, resulting in the formation of immunocomplexes to induce the reorientation of LC molecules so that CA125 can be detected by the change in optical LC texture under crossed polarizers. The anti-CA125 antibody was first immobilized on a DMOAP-coated substrate as the capture molecule for CA125. To avoid false-positive signals, the amount of immobilized anti-CA125 antibody must be controlled to ensure that the homeotropic orientation of the LC film is not affected by the presence of anti-CA125 antibodies at the LC‒DMOAP interface (Figure 6a). As shown in Figure 7, dark optical textures corresponding to homeotropic LC orientation were observed for the spin-coated E7 and HDN films at anti-CA125 antibody concentrations of 10^−7^ and 10^−8^ g/mL, respectively. When the concentration of the anti-CA125 antibody was increased to 10^−6^ g/mL or higher for the E7 film and 10^−7^ g/mL or higher for the HDN film, the optical textures became bright, indicating that the immobilized anti-CA125 antibody alone may reorient LC molecules from the homeotropic to disrupted state, leading to false-positive optical signals in the absence of CA125. As a consequence, in the single-substrate immunoassay based on the E7 and HDN films, the immobilization concentration of the anti-CA125 antibody was limited to 10^−7^ and 10^−8^ g/mL, respectively, which corresponds to the maximal amount of immobilized antibody without background noise. This ensures that the dark-to-bright transition occurring after immunoreaction can be attributed predominantly to CA125 (Figure 6b).

**Figure 8:**
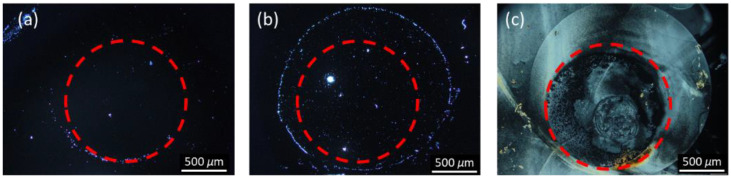
Polarized optical textures of spin-coated E7 films in the presence of the CA125 protein. The nematic LC E7 was spin-coated on DMOAP-coated substrates immobilized with 10^−7^ g/mL anti-CA125 antibody and reacted with CA125 at concentrations of (**a**) 10^−6^, (**b**) 10^−5^, and (**c**) 10^−4^ g/mL. Each red dashed circle represents the area within which the anti-CA125 antibody was immobilized. Scale bar, 500 μm.

**Figure 9:**
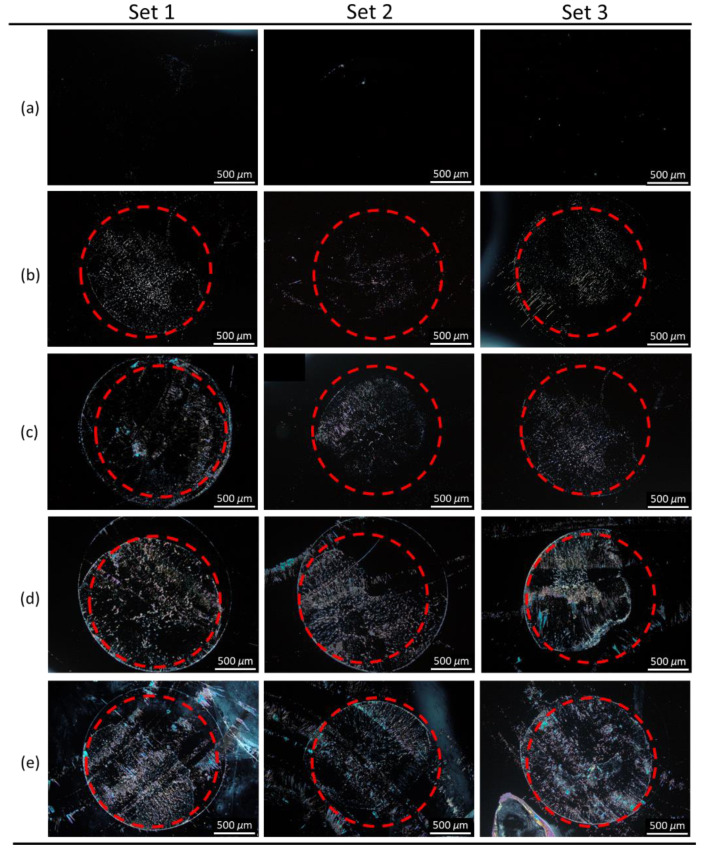
Polarized optical textures of spin-coated HDN films in the presence of the CA125 protein. The nematic LC HDN was spin-coated on DMOAP-coated substrates immobilized with 10^−8^ g/mL anti-CA125 antibody and reacted with CA125 at concentrations of (**a**) 10^−9^, (**b**) 10^−8^, (**c**) 10^−7^, (**d**) 10^−6^, and (**e**) 10^−5^ g/mL. Each red dashed circle represents the area within which the anti-CA125 antibody was immobilized. Sets 1–3 represent results of three independent experiments. Scale bar, 500 μm.

To perform immunodetection of CA125 with the E7 film, 10^−7^ g/mL of the anti-CA125 antibody was immobilized on DMOAP-coated substrates and reacted with different concentrations of CA125, followed by spin-coating of E7. As shown in Figure 8, a red dashed circle is labeled on each micrograph to distinguish the specific binding area (within the circle) immobilized with the anti-CA125 antibody and the nonspecific binding area (outside the circle) where no capture antibody was present. At 10^−^^6^-g/mL CA125, the completely dark texture suggests that the amount of the CA125 immunocomplex was too low to induce reorientation of LC molecules (Figure 8a). When the CA125 concentration was increased to 10^−5^ g/mL, a few bright domains appeared in the specific binding area (Figure 8b). At 10^−4^ g/mL CA125, optical response was observed in both the specific and nonspecific binding areas, which connotes that too much CA125 was present such that, in addition to the formation of immunocomplexes through specific binding, excess CA125 also adsorbed nonspecifically to DMOAP in the area without the anti-CA125 antibody (Figure 8c). The LOD of the LC-based immunoassay with spin-coated E7 film was therefore estimated to be of the order of magnitude of 10^−5^ g/mL CA125. By substituting HDN for E7 as the sensing material in the CA125 immunoassay, the optical texture of the HDN film was dark at 10^−^^9^ g/mL CA125 (Figure 9a), but its brightness gradually increased with CA125 concentration from 10^−8^ to 10^−5^ g/mL (Figure 9b–e). The lower LOD of the HDN film, which was of the order of magnitude of 10^−8^ g/mL CA125, achieved at an anti-CA125 antibody concentration (10^−8^ g/mL) an order of magnitude lower than that for the E7 film can be explained by the higher birefringence of HDN compared with E7. It is ensured that the above-mentioned results specific to immunodetection of CA125 with spin-coated LC films were reproducible at least in three of five sets of experiments, for example, similar optical textures in sets 1–3 for spin-coated HDN LC films in the presence of CA125 as shown in Figure 9. As a comparison, the LOD of a CA125 immunoassay based on sandwiched HDN LC cells was 10^−8^ g/mL CA125 at an anti-CA125 antibody immobilization concentration of 10^−7^ g/mL [38]. It is therefore concluded from the comparable LOD attained with relatively less capture antibody by the LC film that the performance of LC-based optical biosensors can be improved by replacing the sandwiched LC cell with a spin-coated LC film in the single-substrate biosensing platform.

## 4. Conclusions

In summary, a label-free single-substrate biosensor based on a spin-coated LC film was established in this study to achieve lower limit in protein detection and immunoassay. The semi-free LC film was supported by the asymmetric homeotropic anchoring strengths at the LC‒air and LC‒DMOAP interfaces to align LC molecules vertically with their long axes parallel to the substrate normal. Disruption of the ordered orientation of LCs by the presence of biomolecules at the LC‒DMOAP interface leads to dark-to-bright transition of the optical LC texture, giving rise to a high signal-to-background detection signal. Compared to the LC cell, the thickness of the spin-coated LC film is more easily reduced to a thickness smaller than 5 μm by adjusting the spin rate to improve detection sensitivity. When the nematic LC E7 was used in the detection of BSA, the LOD of a 4.2-μm-thick spin-coated E7 film was estimated to be 10^−7^-g/mL BSA, which was two orders of magnitude lower than that of a 4.2-μm-thick sandwiched E7 film in an LC cell. Moreover, by reducing the E7 film thickness to 3.4 μm, the LOD can be further improved to 10^−8^-g/mL BSA. The potential for clinical application of the LC-based single-substrate biosensor was demonstrated with an immunoassay for the cancer biomarker CA125, in which the LOD was determined as 10^−^^5^-g/mL CA125 at an anti-CA125 antibody concentration of 10^−^^7^ g/mL for the E7 film. Substituting HDN, a nematic LC of higher birefringence, for E7 as the sensing medium resulted in a lower LOD of 10^−^^8^-g/mL CA125 with 10^−^^8^-g/mL anti-CA125 antibody immobilized. It is evident from the results of this study that, in addition to the ease of preparation, single-substrate detection with spin-coated LC film offered a new means of signal amplification by reducing film thickness to improve LOD and detection sensitivity. With a wide variety of LCs available in the industries, combined with numerous surface modification and patterning techniques to stabilize the LC film, new possibilities are revealed for the development of more advanced LC-based single-substrate biosensing technologies to extend their practical application.

## Data Availability

The authors confirm that the data supporting the findings of this study are available within the article.
